# Multistate Outbreak of *Campylobacter jejuni* Infections Associated with Undercooked Chicken Livers — Northeastern United States, 2012

**Published:** 2013-11-08

**Authors:** Bradley J. Tompkins, Elisabeth Wirsing, Valarie Devlin, Laura Kamhi, Becky Temple, Keeley Weening, Steffany Cavallo, Latasha Allen, Paul Brinig, Brant Goode, Collette Fitzgerald, Katherine Heiman, Steven Stroika, Barbara Mahon

**Affiliations:** Infectious Disease Section; Environmental Health Div; Public Health Laboratory, Vermont Dept of Health; Bur of Infectious Disease Control, New Hampshire Dept of Health and Human Svcs; Food Safety and Inspection Svc, US Dept of Agriculture; Career Epidemiology Field Officer Program, Office of Public Health Preparedness and Response; Div of Foodborne, Waterborne and Environmental Diseases, National Center for Emerging and Zoonotic Infectious Diseases, CDC

In October 2012 the Vermont Department of Health (VDH) identified three cases of laboratory-confirmed *Campylobacter jejuni* infection in Vermont residents; the isolates had indistinguishable pulsed-field gel electrophoresis (PFGE) patterns. A query of PulseNet, the national molecular subtyping network for foodborne disease surveillance, led to the identification of one additional case each from New Hampshire, New York, and Vermont that had been reported in the preceding 6 months. An investigation led by VDH found that all six patients had been exposed to raw or lightly cooked chicken livers that had been produced at the same Vermont poultry establishment (establishment A). Livers collected from this establishment yielded the outbreak strain of *C. jejuni*. In response, establishment A voluntarily ceased the sale of chicken livers on November 9. A food safety assessment conducted by the U.S. Department of Agriculture’s Food Safety and Inspection Service (USDA-FSIS) found no major violations at the establishment. This is the first reported multistate outbreak of campylobacteriosis associated with chicken liver in the United States. Public health professionals, members of the food industry, and consumers should be aware that chicken livers often are contaminated with *Campylobacter* and that fully cooking products made with chicken liver is the only way to prepare them so they are safe to eat.

## Epidemiologic Investigation

On October 2, 2012, VDH identified two laboratory-confirmed cases of *C. jejuni* infection with indistinguishable *Sma*I and *Kpn*I PFGE patterns (DBRS16.1508 and DBRK02.0049). Patient 1 became ill with diarrhea on September 16 and reported working at a Vermont poultry establishment (establishment A); his food history was unremarkable and did not include any products from establishment A. His work duties involved handling live and slaughtered chickens and turkeys. Patient 2 also became ill on September 16 and was hospitalized 4 days later. He reported eating a charcuterie (meat platter) appetizer and rabbit entree at a Vermont restaurant (restaurant A) 2 days before his illness onset. The charcuterie included a mousse made from chicken livers produced at establishment A.

Patient 3 became ill on September 20; she reported eating the same menu items at restaurant A 1 day after patient 2. The *C. jejuni* isolate from her stool specimen yielded a PFGE pattern indistinguishable from the outbreak strain.

A retrospective cohort study of patrons who dined at restaurant A within 2 days of the patients with confirmed *C. jejuni* infection was conducted. Contact information was obtained from the restaurant’s reservation list. A total of 43 diners were contacted in addition to patients 1 and 2; one diner declined to participate in the study. Diners were asked what they ate and whether they experienced any diarrhea in the subsequent 10 days. No additional diners reported diarrhea; therefore, no probable cases were identified.

Nineteen menu items were analyzed for a statistical association with illness by calculating relative risks (RR). A value of 0.5 was added to all cells in 2×2 tables that contained a zero. Consumption of only two menu items showed a statistically significant relative risk of illness: charcuterie that included chicken liver mousse (RR = 52.5, 95% confidence interval [CI] = 3.0–914.8) was consumed by three patrons, and rabbit (RR = 33.3, CI = 1.8–613.5) was consumed by five. Although limited by a small sample size (resulting in wide CIs), the higher relative risk associated with consuming charcuterie as well as isolation of the outbreak strain of *C. jejuni* in a worker at establishment A, where the chicken livers were produced, focused the investigation on chicken livers.

PulseNet identified a fourth Vermont isolate indistinguishable by PFGE from the outbreak strain. Patient 4 had not reported eating chicken livers when originally interviewed in June 2012 by VDH, which investigates all reports of campylobacteriosis. But upon reinterview as part of this investigation, patient 4 reported eating pan-fried chicken livers at another Vermont restaurant (restaurant B) several days before becoming ill. An interview with restaurant B staff members revealed that establishment A was the source of their chicken livers in June 2012.

VDH notified other New England states in which establishment A products were distributed and requested information on any patients with *C. jejuni* infection who reported consumption of chicken livers or whose isolates had PFGE patterns indistinguishable from the outbreak strain. PulseNet identified one April 2012 isolate from a New Hampshire resident (patient 5) with a *Sma*I PFGE pattern indistinguishable from the outbreak strain. The New Hampshire Department of Health and Human Services performed additional PFGE testing on this isolate using *Kpn*I and found the pattern to be indistinguishable from the outbreak strain. Patient 5 reported purchasing raw chicken livers from a New Hampshire grocery store and cooking them to medium rare at home for herself and family members, one of whom was a female New York resident (patient 6) who had been hospitalized in April 2012 with *C. jejuni* infection. Following notification of the outbreak, New York state analyzed the isolate from patient 6 and found its PFGE pattern indistinguishable from the outbreak strain.

The six patients ranged in age from 19 to 87 years (median: 53.5 years); three were female. Two were hospitalized, but all six had recovered by the time of their initial interviews.

## Environmental Investigation

VDH inspected restaurants A and B. Both restaurants passed inspection with no critical violations noted. Stool specimens collected from all eight food handlers at restaurant A did not yield *Campylobacter.* Both restaurants confirmed that they received fresh chicken livers from establishment A and froze them until needed. Interviews with both chefs revealed that chicken livers were lightly cooked to maintain their texture. In accordance with VDH health regulations for food service establishments, the menu at both restaurants contained the required general consumer advisory regarding the increased risk of foodborne illness from consuming raw or undercooked poultry. VDH regulations do not require that the menus at food service establishments identify specific food items that are potentially hazardous and served raw or undercooked; therefore, the chicken liver dishes at restaurants A and B were not individually labeled as lightly cooked.

The New Hampshire Department of Health and Human Services reviewed grocery store records and, based on the purchase date reported by patient 5, identified establishment A as the source of the livers that patients 5 and 6 consumed.

USDA-FSIS conducted a food safety assessment at establishment A and found that the establishment had a well-designed food safety system, which included application of antimicrobial cleaners to the poultry products. When observed during the assessment, these cleaners were used as intended to reduce contamination on the surfaces of all poultry carcasses and parts. The assessment revealed no extrinsic factors, such as cross contamination, that would likely cause the chicken livers to be tainted.

## Laboratory Investigation

Frozen chicken livers collected from restaurant A were sent to the VDH laboratory, where they were minced into 13 25-gram subsamples and enriched in accordance with the instructions for the *Campylobacter* immunoassay. Two of the 13 subsamples screened with the immunoassay for the presence of *Campylobacter* gave positive results, but the pathogen could not be recovered in culture.

VDH then collected fresh chicken livers directly from establishment A and delivered them to the VDH laboratory, where they were processed in accordance with testing instructions. *C. jejuni* was recovered from these chicken livers, and one isolate had PFGE patterns indistinguishable from the outbreak strain.

Additional characterization of the six human isolates and one chicken liver isolate by antimicrobial susceptibility testing identified this outbreak strain as susceptible to eight of nine antimicrobials tested on the CDC National Antimicrobial Resistance Monitoring System panel, but resistant to tetracycline. Multilocus sequence typing identified the outbreak strain as sequence type 1212.

Establishment A was notified of the results of the investigation on November 9. The establishment ceased selling chicken livers that same day.

### Editorial Note

*Campylobacter* is the third-leading cause of bacterial foodborne illness in the United States (1), and poultry exposure is a well-recognized risk factor for infection. Poultry-associated campylobacteriosis is the pathogen-food pair estimated to be responsible for the greatest burden of foodborne disease in the United States (2). Despite this, documented outbreaks of *Campylobacter* are relatively rare, with only 1.9% of all foodborne outbreaks reported to CDC’s National Outbreak Reporting System attributed to this pathogen (3). Rarer still are documented *Campylobacter* outbreaks caused by poultry livers. Between 1997 and 2008, five such outbreaks were reported, but only two of these reports confirmed poultry livers as the vehicle (4). Unlike the outbreak reported here, none of these previous outbreaks were multistate, nor did any previous investigation confirm livers as the outbreak source using laboratory evidence.

Outbreaks of *Campylobacter* infections linked to chicken livers have been reported in the United Kingdom (5) and Australia (6). Since 2007, England and Wales have seen a significant increase in the proportion of *Campylobacter* outbreaks linked to chicken livers used in pâté (7).

What is already known on this topic?

*Campylobacter* is a common cause of bacterial foodborne illness, but documented outbreaks caused by the pathogen are relatively rare in the United States. *Campylobacter* outbreaks caused by consumption of undercooked chicken liver have been well documented overseas.

What is added by this report?

Chicken livers from a Vermont poultry establishment were implicated as the cause of an outbreak of *Campylobacter jejuni* infection in the northeastern United States in 2012. Six patients were identified; two were hospitalized. Five patients were exposed through consumption of chicken livers; one patient worked at the establishment where the livers were produced. Raw livers yielded the outbreak strain of *C. jejuni.* Inspection of the poultry producer and two restaurants associated with three of the cases revealed no significant defects in food storage or preparation except that chicken livers were not thoroughly cooked. In response to the investigation, the poultry producer permanently halted the sale of this product.

What are the implications for public health practice?

Public health officials, food industry personnel, and consumers should be aware that chicken livers often are contaminated internally with *Campylobacter* and cannot be made safe to eat without being fully cooked. Pulsed-field gel electrophoresis of *Campylobacter* isolates can be a helpful tool for investigating suspected outbreaks and might assist with case finding, which could lead to a more accurate assessment of the scope of an outbreak.

These outbreaks should not come as a surprise, given that previous studies have shown that 77% of retail chicken livers are contaminated with *Campylobacter* (8) and that, when contamination is present, it is usually in internal tissues, as well as on the surface (9). The Food and Drug Administration food code states that poultry must reach an internal temperature of 165°F (73.9°C) for at least 15 seconds. Studies outside the United States have found that in order for chicken livers to be free of *Campylobacter* they must be heated to internal temperatures in excess of 158°F (70°C) and held at that temperature for 2–3 minutes (9). In this investigation, the livers were found to be intentionally cooked lightly to maintain a desired texture and taste. This practice might be common, particularly when preparing chicken livers for use in a mousse or pâté. A popular recipe for this dish instructs readers to cook “until the livers are just stiffened, but still rosy inside” (10).

Although USDA-FSIS found that establishment A applied antimicrobial cleaners to the livers, these efforts only affect the external surfaces of chicken livers, and because *Campylobacter* contamination can be internal, the safety of undercooked chicken livers cannot be assured. Ultimately, establishment A stopped selling chicken livers.

Vermont is one of the few states that investigates all reported cases of campylobacteriosis and performs PFGE on all *Campylobacter* isolates submitted to the VDH laboratory. This strategy, along with the combined efforts of state and federal partners, enabled the timely detection of the outbreak and identification of the source. This investigation emphasizes the potential risk for *Campylobacter* infection from consumption of undercooked chicken livers and the potential for this pathogen-food pair to cause outbreaks in the United States.
